# Strategies in Surgical Decompression for Thyroid Eye Disease

**DOI:** 10.1155/2020/3537675

**Published:** 2020-09-09

**Authors:** Anny M. S. Cheng, Yi-Hsuan Wei, Shu-Lang Liao

**Affiliations:** ^1^Florida International University, Herbert Wertheim College of Medicine, Florida, USA; ^2^Department of Surgery, Miller School of Medicine, University of Medicine, Miami, Florida, USA; ^3^Department of Ophthalmology, National Taiwan University Hospital, Taipei, Taiwan; ^4^School of Medicine, National Taiwan University, Taipei, Taiwan

## Abstract

Surgical management of thyroid eye disease- (TED-) associated morbidity has been plagued by the complex interplay of different operative techniques. Orbital decompression is the well-recognized procedure for disfiguring exophthalmos and dysthyroid optic neuropathy (DON). There are numerous published techniques described for the removal of the orbital bone, fat, or a combination. The diverse studies are noncomparative as they include different indications, stages of disease, and methods of evaluation. Thus, it is difficult to conclude the most efficient decompression technique. To obtain effective and predictable results, it is therefore important to propose a logical and acceptable clinical guideline to customize patient treatment. Herein, we developed an algorithm based on the presence of DON, preoperative existing diplopia, and severity of proptosis which were defined by patient's disabling symptoms together with a set of ocular signs reflecting visual function or cosmesis. More specifically, we aimed to assess the minimal but effective surgical technique with acceptable potential complications to achieve therapeutic efficacy. Transcaruncular or inferomedial decompressions are indicated in restoring optic nerve function in patients with DON associated with mild or moderate to severe proptosis, respectively. Inferomedial or fatty decompressions are effective to treat patients with existing diplopia associated with mild or moderate to severe proptosis, respectively. Fatty or balanced decompressions can improve disfiguring exophthalmos in patients without existing diplopia associated with mild to moderate or severe proptosis, respectively. Inferomedial or 3-wall decompressions are preferred to address facial rehabilitation in patients associated with very severe proptosis but without preoperative diplopia.

## 1. Introduction

Thyroid eye disease (TED) is a complex autoimmune disease closely associated with orbital inflammation that has been puzzling for centuries [1]. Progress has been made in the understanding of the pathogenesis of TED. Orbital fibroblast is recognized as a prime target [2]; the recent finding that extraocular muscle (EOM) can be vitally involved is also important [3]. Cytokines play important roles in orbital inflammation during the active phase [4], with subsequent tissue remodeling and fibrosis during the inactive phase [5]. While the orbital fibroblasts secrete hydrophilic hyaluronan in response to cytokines [6] and a subgroup of fibroblasts differentiate into mature adipocytes [7], EOM initiates and participates in a self-perpetuation of inflammation in TED [8]. These cellular changes lead to the characteristic enlargement of EOM and expansion of orbital fat of patients with TED, with a predominance of one or the other in some [9]. The discrepancy between the increased volume of the swollen tissues and the fixed volume of the bony orbit results in proptosis and orbital congestion that induce periorbital swelling, increased lid aperture, corneal exposure, and compressive optic neuropathy which consequently lead to significant visual morbidity.

Our understanding of the orbital pathophysiology of TED points to several potential therapeutic targets. Proper management of TED depends on evaluation based on clinical activity and severity. For active inflammation, several treatment strategies focus on immune suppression are available. The mainstay of treatment is corticosteroids to potentially diminish or shorten the acute inflammatory phase; steroid sparing options include orbital irradiation, immunosuppressive drugs inhibiting T cell, B cells, cytokines, mono- or polyclonal antibodies against tumor necrosis factor receptor, insulin-like growth factor1 receptor, thyrotropin receptor, and CD40 or PI3K intracellular pathway are also showing promising results (reviewed in [10]). However, some patients do not experience significant clinical improvement and may develop inflammatory sequel despite anti-inflammatory therapies. When the disease progresses to fibrotic inactive TED, the clinical course is stable to proceed with surgical rehabilitation.

Surgical treatment in TED is orbital decompression in addressing rehabilitation like proptosis and tissue scarring along with squint correction and lid repair in chronological order. Orbital decompression can also be an urgent procedure in vision-threatening conditions like dysthyroid optic neuropathy (DON) to relieve pressure on the orbital apex and improve vascular/axonal flow within the optic nerve (reviewed in [11]). Various techniques and approaches for orbital decompression exist. Orbital decompression has been described as one-, two-, and three-wall bony decompressions with or without orbital fat removal, solely fatty decompression, and with or without the use of the endoscope [12]. However, there is no consensus on the most efficient intervention. The current surgical technique mostly relates to surgeons' personal preferences and varies geographically [13]. As no single approach has been adopted as the gold standard, it is helpful to generate a practical algorithm to guide and tailor the specific surgical approach to an individual patient. This report offers a tailor-made approach focusing on orbital decompression by the monitoring of the presence of DON and severity of proptosis.

## 2. Principle of DON Treatment

TED with DON is mainly a result of crowding around the orbital apex with nerve compression secondary to expanded extraocular muscles [14] or some caused by optic nerve stretching without a crowded apex [15]. In a British study of 71 eyes of 49 patients with DON, most patients received initial corticosteroid therapy yet almost 50% required surgical orbital decompression [16]. A large American study of 163 eyes of 104 patients with DON received combined corticosteroid and orbital radiotherapy has reported that among the initially 95% successfully treated patients, ultimately 36.7% of them underwent elective surgery, including orbital decompression during the inactive phase of TED [17]. Another German study of 46 eyes of 25 patients with DON showed that the mild cases with better initial visual acuity (logMAR 0.3) responded well to steroid treatment but not the cases with an impaired initial visual acuity (logMAR 0.6) [18]. Surgical decompression among the first-line medical therapy-resistant eyes, however, was effective to reduce the pressure on the optic nerve by lessening the volume of the orbital content, decreasing the ongoing inflammation, and thereby preserving the optic nerve function.

Taking together, orbital decompression surgery is best performed in the inactive phase of TED for rehabilitation, but decompression may also be required in the active phase for cases of DON that are refractory to medical treatment [18, 19].

## 3. Clinical Decision Making in Surgical Decompression for DON

Orbital bony wall decompression was first performed in the1930s for patients with DON [20]. The techniques for orbital decompression in the management of TED with DON have continued to evolve in the last 30 years. Compression of the optic nerve or restriction of its blood supply by enlarged EOM or expanded soft tissue or fat is currently the most widely accepted mechanism of DON [14]. Intriguingly, marked proptosis is not always present in patients with DON [21]. Although the severity of proptosis may not proportionally correlate with the severity of neuropathy, proptosis could increase the orbital pressure and precipitate DON. Regarding the clinical assessment of patients with DON, proptosis measurement is still an important parameter.

DON has been managed with bony decompression by various techniques. Early studies suggest that (infero)medial wall decompression is an effective surgical option for DON. Several surgical methods, including the transantral, transcutaneous, transconjunctival, endonasal, and transcaruncular approaches, have been adopted [22–26] to decompress particularly the posterior medial and inferior walls near the optic nerve apex. Specifically, the transcaruncular approach to the medial orbit provides wide exposure and safe access to the medial extraperiosteal space. This approach allows quick and direct visualization of the entire medial and inferomedial orbit. With this approach, removal of the bone along the medial wall including an assessment to the anterior and posterior ethmoid arteries, the sphenoid sinus, and inferior apex is easy to accomplish. Several studies demonstrated significant improvement in all parameters of the optic nerve function after the transcaruncular approach decompression [27–30].

In the Taiwanese studies of 41 eyes of 23 patients and 38 eyes of 22 patients with DON that underwent transcaruncular approach decompression, the proptosis reductions were 3.4 ± 1.2 mm [31] and 3.7 ± 1.6 mm [29], respectively. In contrast, studies reported a more significant reduction of proptosis ranging from 4 mm to 6 mm in inferomedial orbital decompression via the transconjunctival, transantral, transcutaneous, or endonasal approach [31–34]. The explored area through the transcaruncular approach is the total medial orbital wall and only part of the inferior orbital wall. Therefore, transcaruncular orbital apex decompression removed less total bony area than that removed from inferomedial orbital decompression via the transconjunctival, transantral, transcutaneous, or endonasal approach. Importantly, the aim of managing the DON is to release the compression around the optic nerve, while the posterior medial wall removal is the most effective in relieving orbital apex pressure. Specifically, the transcaruncular approach allows for the entire medial wall exposure which has the best visualization than traditional transconjunctival, transantral, or transcutaneous inferomedial wall decompression. Furthermore, the transcaruncular approach targets at the orbital apex bone which is more posterior than all other approaches in inferomedial wall decompression.

As the transcaruncular approach preserves the maxillo-ethmoidal strut, it lowers the risk of new-onset postoperative diplopia [24, 27, 29]. The diplopia rate ranged from 20% to 38% in the transcaruncular approach but high up to 70% in inferomedial decompression via transantral [22, 31, 32] or 80% via endoscopic transnasal approach [25, 35]. Although recent endoscopic transnasal approach showed less postoperative diplopia [36], this approach is unfamiliar to most ophthalmic surgeons.

Fatty decompression has been shown for the treatment of DON [37]; however, it was mostly applied in patients with more fat compartment enlargement but modest EOM enlargement. Removal of the lateral wall can sufficiently decompress the orbital wall [38] but may not extend as far posteriorly as the medial wall to decompress the orbital apex effectively for the treatment of DON. Another procedure such as three-wall decompression is effective in orbital apex reduction; however, new-onset diplopia and orbital complications are more common [34].

**Figure 1:**
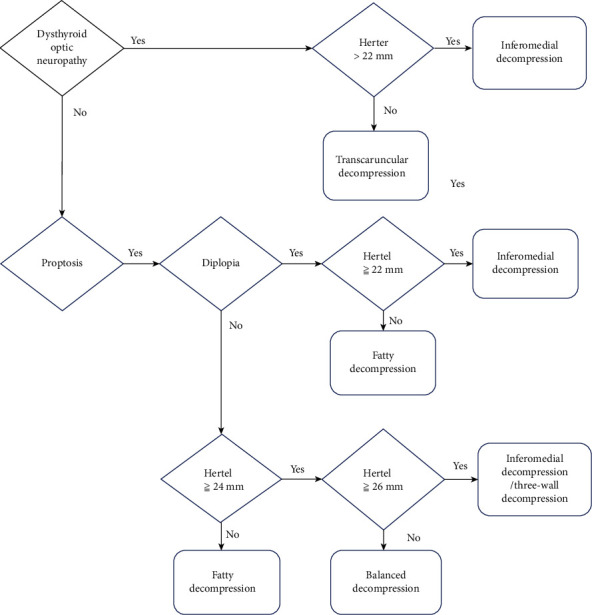
Summary of our proposition for a surgical strategy in the management of dysthyroid optic neuropathy and proptosis in thyroid eye disease.

Taken together, we suggest that for patients with DON and mild proptosis (Hertel < 22 mm), it is reasonable to relieve optic nerve compression by transcaruncular medial wall decompression without much reduction of proptosis. For patients with DON and moderate to severe proptosis (Hertel > 22 mm), we favor the use of inferomedial wall decompression via transconjunctival, transantral, or transcutaneous approaches (Figure 1).

## 4. A Practical Algorithm for Surgical Decompression in Disfiguring Exophthalmos

Rehabilitative orbital decompression surgery is performed during the stable stage of TED for cosmetic rejuvenation [39]. Disfiguring exophthalmos in TED remains a therapeutic challenge as to date; there are abundant published data on surgical orbital decompression, mainly retrospective and case series reports that were heterogeneous in patients and decompression techniques but few comparative studies. Decompression can be achieved via typical areas including the floor, the medial wall, the inferomedial wall, the lateral wall, and the fat compartment. It is well established that every bone can be removed via various surgical approaches with targeting expanded orbital volume. Evidence showed that the type of approach (such as transconjunctival, transantral, or transcutaneous) does not significantly affect overall surgical outcomes [34]. Instead, a superior reduction of proptosis with a low complication rate is the key to address facial rehabilitation aimed at reducing proptosis, restoring function, and enhancing appearance to improve quality of life. There is still no evidence-based conclusion regarding which method offers optimum decompression with the lowest complication rate. Taking this concern into consideration, we propose to adopt an algorithm in a logical and stepwise manner that helps to define acceptable clinical standards and aid surgeons in decision-making to tailor each unique technical consideration and potential complications to the patient with disfiguring exophthalmos.

### 4.1. Step 1: Measurement of Proptosis

In the preoperative assessment of the stable disfiguring exophthalmos in TED, the first step is to determine the extent of proptosis. Orbital decompression surgery, with the removal of one or more orbital walls, reduces proptosis by allowing voluminous orbital tissue prolapse into the new bony defects or sinuses [40]. For decompression with different wall involvement, there is a wide range of reported proptosis reductions. Any combination of the medial, inferior, or lateral walls can be targeted and the number of walls removed usually determined the amount of proptosis reduction.

While isolated floor decompression is rarely performed, isolated medial wall decompression with or without endoscopic approach has reported proptosis reduction of 2.5-3.1 mm or 1-4 mm, respectively (reviewed in [41]). Single lateral wall decompression achieved decompression effects of 2.7-4.8 mm [42, 43]. When combined medial with the lateral wall as balanced decompression, larger proptosis reduction can be achieved between 4 and 5.5 mm [44–46]. As reported by different publications, combined medial with floor wall as inferomedial wall decompression has a large amount of proptosis reduction ranging from 4 to 6 mm [33, 40, 47]. The 3-wall decompression consisting of the medial wall, floor, and the lateral wall has reported a very large proptosis reduction in the range of 4.5-7.5 mm [48–50]. Orbital bony decompression may or may not combine fat removal. For solely fatty decompression, the efficacy of proptosis reduction has been reported as 3.5-5.9 mm [51–54] in the short-/intermediate-term or 4.2 ± 1.4 mm in the long-term follow-up [3].

### 4.2. Step 2: Preoperative Diplopia Present or Not

Diplopia is another disabling symptom other than exophthalmos for patients with TED. The impairment of motility of EOM is caused by inflammation that subsequently results in muscle fibrosis and fatty degeneration [1]. Since diplopia is the most common postoperative complication of decompression surgery [55], it is important to measure the misalignment in the primary and reading positions in preoperative assessment. Of note, a study showed that patients with preoperative diplopia were more prone to develop primary gaze diplopia after orbital decompression, independently of the surgical technique [56]. Diplopia can worsen or newly develop because of muscle displacement in changes of orbital anatomy after orbital decompression. Therefore, other than the severity of proptosis, the surgeon should consider the presence of preoperative diplopia when discussed preoperatively with the patient about the balance of the risk of postoperative diplopia and the rehabilitative beneficial effect.

Generally, patients who underwent isolated medial wall, inferomedial, or balanced decompression have the comparable newly onset diplopia rates reported at 14-19% [41], 10-35% [40, 47], and 10-20% [47, 56], respectively. The 3-wall decompression, with the addition of one-wall decompression, led to a higher incidence of new diplopia of 57% [50]. The risk of newly onset diplopia in lateral and fatty decompressions tends to be low in the range of 0-6% [28, 57, 58] and 3.3% [3], respectively.

### 4.3. Step 3: Surgical Algorithm (Figure 1)

#### 4.3.1. Presence of Preoperative Diplopia with Mild Proptosis (Hertel < 22 mm): Fatty Decompression

To avoid adding on disabling complications correlated to the surgery, decompression with low newly onset diplopia rate is preferred. Both fat and lateral decompressions have a low rate of newly onset diplopia. Decompression involved the lateral wall; however, it is relatively proximity to cranial with a cerebrospinal fluid leakage rate of 3-7% [45, 46, 50], whereas none was reported in fatty decompression. As fatty decompression is effective but potentially limited in treating very large proptosis eyes [3], fatty decompression is indicated for patients with preexisting diplopia and mild proptosis

#### 4.3.2. Presence of Preoperative Diplopia with Moderate to Severe Proptosis (Hertel ≥ 22 mm): Inferomedial Decompression

2-wall including balanced or inferomedial decompression or 3-wall decompression is more effective in the correction of moderate to severe proptosis. The concern that 3-wall surgery may increase the incidence of postoperative newly onset diplopia and balanced decompression may have the possible serious complication of a cerebrospinal fluid leakage that makes inferomedial decompression a better option for patients with preexisting diplopia and moderate to severe proptosis

#### 4.3.3. Absence of Preoperative Diplopia with Mild to Moderate Proptosis (Hertel < 24 mm): Fatty Decompression

When a patient is without an initial presentation of diplopia, we focus mainly on the extent of correction of proptosis in different surgical techniques. As fat, 2-wall, or 3-wall decompression with reported proptosis reduction rates of 3.5-5.9 mm, 4-6 mm, and 4.5-7.5 mm, respectively, are effective for moderate to severe proptosis, it is appropriate for patients with moderate proptosis but no preoperative diplopia undergo fatty decompression to avoid postoperative newly onset diplopia

#### 4.3.4. Absence of Preoperative Diplopia with Severe Proptosis (Hertel ≥ 24 mm but < 26 mm): Balanced Decompression.

Both 2-wall or 3-wall decompressions are comparative for severe proptosis. Taking into account that more proptosis reduction can be achieved with more wall removal [47], the 3-wall decompression, which may increase the incidence of complication, is reserved for correction of very severe proptosis. Compared with the two 2-wall decompressions, inferomedial decompression may have slightly higher proptosis regression and newly onset diplopia than balanced decompression. Therefore, we recommend balanced decompression for patients with severe proptosis but no preoperative diplopia

#### 4.3.5. Absence of Preoperative Diplopia with Very Severe Proptosis (Hertel ≥ 26 mm): Inferomedial Decompression or 3-wall Decompression.

Regardless of the high complication rate of postoperative diplopia, 3-wall decompression is effective for very severe proptosis. Inferomedial decompression achieves a comparable reduction in proptosis but a lower rate of diplopia. Hence, we suggest either inferomedial or 3-wall decompression for patients with very severe proptosis but no preoperative diplopia

## 5. Conclusion

In summary, surgical orbital decompressions are a recognized procedure for the management of some patients with TED. We generate a practical algorithm based on the presence of DON, diplopia, and the severity of proptosis to guide surgical treatment. For DON refractory to immunosuppression in the active stage, transcaruncular approach decompression has good results in restoring optic nerve function in patients with mild proptosis, whereas inferomedial decompression is more suitable for those with moderate to severe proptosis. For disfiguring exophthalmos associated with diplopia in the inactive stage, fatty decompression is the surgery of choice for patients with mild proptosis, whereas inferomedial decompression is indicated for those with moderate to severe proptosis. For disfiguring exophthalmos without existing diplopia, fatty decompression and balanced decompression are effective for patients with mild to moderate and severe proptosis, respectively. Lastly, inferomedial or 3-wall decompression is preferred for patients with very severe proptosis but without preoperative diplopia to address facial rehabilitation. Given these considerations, surgeons can perform a custom decompression by indicated parameters.

## Data Availability

Data supporting the results can be found to publicly archived datasets.
